# Disease characteristics and treatment patterns of Chinese patients with metastatic colorectal cancer: a retrospective study using medical records from China

**DOI:** 10.1186/s12885-020-6557-5

**Published:** 2020-02-18

**Authors:** Ruihua Xu, Wei Wang, Bo Zhu, Xiaoyan Lin, Dong Ma, Lingjun Zhu, Qingchuan Zhao, Yongzhan Nie, Xiaohong Cai, Qi Li, Weijia Fang, Hongyan Li, Ning Wang, Yun Chen, Cike Peng, Honghao Fang, Lin Shen

**Affiliations:** 1 Sun Yat-sen University Cancer Center, No. 651 Dongfeng East Road, Yuexiu District, Guangzhou, 510060 China; 2 The First People’s Hospital of Foshan, No. 81 North Lingnan Road, Chancheng District, Foshan, 528000 China; 3 Xinqiao Hospital, Third Military Medical University, No. 83 Xinqiaozheng Street, Shapingbei District, Chongqing, 400037 China; 4 Fujian Medical University Union Hospital, No. 29 Xinquan Road, Gulou District, Fuzhou, 350001 China; 5 Guangdong General Hospital, No.106 Zhongshan Road, Yuexiu District, Guangzhou, 510245 China; 6 Jiangsu Province Hospital, No.300 Guangzhou Road, Gulou District, Nanjing, 210029 China; 7 Xijing Hospital, Fourth Military Medical University, No.15 West Road, Xincheng District, 710032, Xi’an, Changle, China; 8 Sichuan Cancer Hospital and Institute, No.55 Sectional 4, South Renmin Road, Wuhou District, Chengdu, 610015 China; 9 Shanghai General Hospital, No.100 Haining Road, Hongkou District, Shanghai, 200080 China; 10 The First Hospital of Zhejiang Province, No.79 Qingchun Road, Shangcheng District, Hangzhou, 310002 China; 11 Eli Lilly and Company China Affiliate, No. 288 Shi Men Yi Lu, Jing’an District, Shanghai, 200041 China; 12 IQVIA, No. 968 West Beijing Road, Jing’an District, Shanghai, 200063 China; 13 Beijing Cancer Hospital, No. 52 Fucheng Road, Haidian District, Beijing, 100142 China

**Keywords:** Treatment patterns, Colorectal cancer, Medical records, Real-world evidence, China

## Abstract

**Background:**

Colorectal cancer (CRC) is the third most prevalent cancer in China but few large-scale studies were conducted to understand CRC patients. The current study is aimed to gain a real-world perspectives of CRC patients in China.

**Methods:**

Using electronic medical records of sampled patients between 2011 and 2016 from 12 hospitals in China, a retrospective cohort study was conducted to describe demographics and disease prognosis of CRC patients, and examine treatment sequences among metastatic CRC (mCRC) patients. Descriptive, comparative and survival analyses were conducted.

**Results:**

Among mCRC patients (3878/8136, 48%), the fluorouracil, leucovorin, and oxaliplatin (FOLFOX) and other oxaliplatin-based regimens were the most widely-used first-line treatment (42%). Fluorouracil, leucovorin, irinotecan (FOLFIRI) and other irinotecan-based regimens dominated the second-line (40%). There was no a dominated regimen for the third-line. The proportion of patients receiving chemotherapy with targeted biologics increased from less than 20% for the first- and second- lines to 34% for the third-line (*p* < 0.001). The most common sequence from first- to second-line was from FOLFOX and other oxaliplatin-based regimens to FOLFIRI and other irinotecan-based regimens (286/1200, 24%).

**Conclusions:**

Our findings reflected a lack of consensus on the choice of third-line therapy and limited available options in China. It is evident o continue promoting early CRC diagnosis and to increase the accessibility of treatment options for mCRC patients. As the only nationwide large-scale study among CRC and mCRC patients before more biologics became available in China, our results can also be used as the baseline to assess treatment pattern changes before and after more third-line treatment were approved and covered into the National Health Insurance Plan in China between 2017 and 2018.

## Background

Colorectal cancer (CRC) is the third most common cancer in China, with 370,000 new cases in year 2014, comprising 9.73% of all cancers [1]. CRC is also one of the leading causes of cancer deaths in China. Risks of CRC increase with age, especially after age 35, and reach a peak among people aged 80–84 years old [2]. The age-standardized incidence rate by Chinese standard population (ASIRC) was estimated to be 14.20 per 100,000 in 2012, increasing to 17.45 per 100,000 in 2013 and to 17.76 per 100,000 in 2014 [1, 2]. Besides age, there has been evidence that a diet high in fats and low in fruits and vegetables increases the risk of developing CRC [3, 4]. Because of the rapidly aging population and in the increasing fat intake in China, CRC incidence is expected to continue increasing. Moreover, the National Central Cancer Registry (NCCR) showed that prognosis of CRC was much poorer in China compared with developed countries [5].

Despite the significant disease burden, there is limited information on CRC patient characteristics and disease patterns in China. The NCCR synthesizes data collected from local registries in China and reports basic statistics such as incidence and mortality by key risk groups at the national level [6]. Population-based studies were usually conducted in particular geographic regions [7–10], among a specific group of patients [8], or out-of-date [11]. For CRC patients with metastatic colorectal cancer (mCRC), treatments can be complex. As multiple chemotherapeutic and targeted biologic agents emerged, treatment patterns and sequences for mCRC patients have significantly evolved over the past decade [12]. Several studies have reported the complex and changing treatment pattern among mCRC patients in the United States [13–16], Canada [17], and some European countries [18, 19]. However, there is a lack of key real-world evidence on the clinical characteristics and treatment patterns of CRC/mCRC patients in China.

Thus, this study was designed to describe baseline characteristics of CRC and mCRC patients, to investigate prognosis in CRC patients, as well as to understand treatment patterns and sequences in mCRC patients using a multi-center oncology database [20]. Findings from this study can be used as evidence to inform clinical management of CRC and mCRC patients.

## Methods

### Data source

This study analyzed data drawn from a multi-center oncology database, which gathered information from electronic medical records (EMRs) of multiple tertiary hospitals in China. With a large volume of patient-level data of patient sample and a wide geographic coverage, this database provides a platform for conducting a retrospective database study among CRC and mCRC patients. A total of 12 tertiary hospitals from eight provinces were selected across China. Data between January 1, 2011 and September 30, 2016 were extracted, including information on patient baseline characteristics and detailed diagnosis and treatment-related information during each inpatient visit.

### Study population

Patients with primary diagnosis dates after January 1, 2011 and aged 18 years old and above at primary diagnosis were included. Primary diagnosis dates (baseline) were defined as the first clinical or pathological diagnosis dates recorded in the selected hospital’s EMRs, whichever occurred earlier. If patients were previously diagnosed with CRC outside the selected hospitals, the initial diagnosis dates, if available, were used as the primary diagnosis dates. As a previous phase III clinical trial suggested that the maximum period of the 3rd line treatment usage was nine months [21], in this study, patients who started third-line chemotherapy after January 1, 2016 were excluded to allow a minimum nine-month observation period for the entire third-line chemotherapy to be documented.

Patients with mCRC, as a subgroup of CRC patients, included those who were classified as TNM stage IV [22] at primary diagnosis, and those whose tumor metastasized before the database lock (September 30, 2016).

### Study variables

Patient demographic and clinical characteristics at primary diagnosis were analyzed for the CRC and the mCRC subgroup. As the first appearance of metastases was not recorded in EMRs, metastases were defined by the adoption of palliative chemotherapy. Recurrence rates were assessed at 1-year, 2-year and 3-year post-index dates and compared by TNM stage and primary tumor site. Disease-free survival was defined as the time between the primary diagnosis date and the first documentation of recurrent of local or regional tumor, or deaths or the last record date in the EMR database, whichever occurred earlier. Treatment patterns of first-, second- and third-line palliative chemotherapy were also analyzed, with regimens and cycles reported. Chemotherapeutic lines were determined by physicians and recorded in EMRs.

### Statistics

For continuous variables, mean, standard deviation (SD), median, interquartile range (IQR), minimum (min) and maximum (max) values were presented as appropriate; for categorical variables, number of missing values, frequency distribution and percentage were presented. Missing data were not included in percentage calculations. Chi-square tests were used to compare categorical variables and Kruskal-Wallis tests for continuous variables. Recurrence rates were compared by TNM stage and primary tumor site using Fisher’s exact tests instead of Chi-square tests due to a smaller sample size. Cumulative probabilities of disease-free survival by TNM stage were estimated using the Kaplan-Meier method, and comparisons were performed with the log-rank test. All statistical analyses were performed using STATA version 14.0 (Stata Corporation, Texas, USA). Two-sided tests with a significance level of 0.05 were applied.

## Results

### Patient flow

**Fig. 1:**
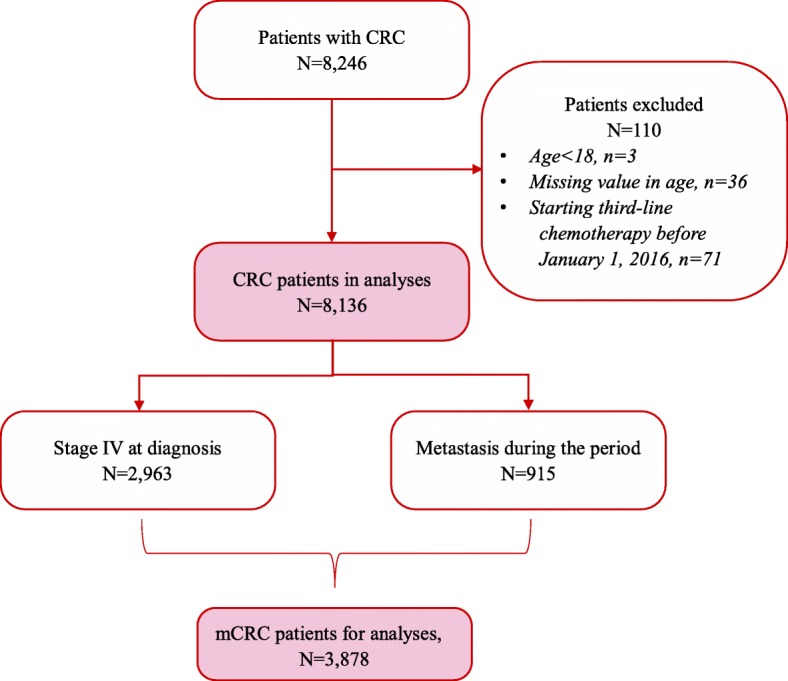
Patient flow

Data of 8246 CRC patients identified from the selected hospitals during the study period were extracted and screened. Among them, 39 patients had missing values or were under 18 years old at primary diagnosis, and 71 patients started third-line palliative chemotherapy after January 1, 2016. After excluding these 110 patients, 8136 CRC patients were included (Fig. 1). Over one-third (2963/8136, 36%) of these patients were at TNM stage IV at primary diagnosis, and an additional 915 (11%) patients metastasized during the observation period. Thus, 3878 (47% of 8246) mCRC patients were identified.

### Baseline characteristics for CRC patients

**Fig. 2:**
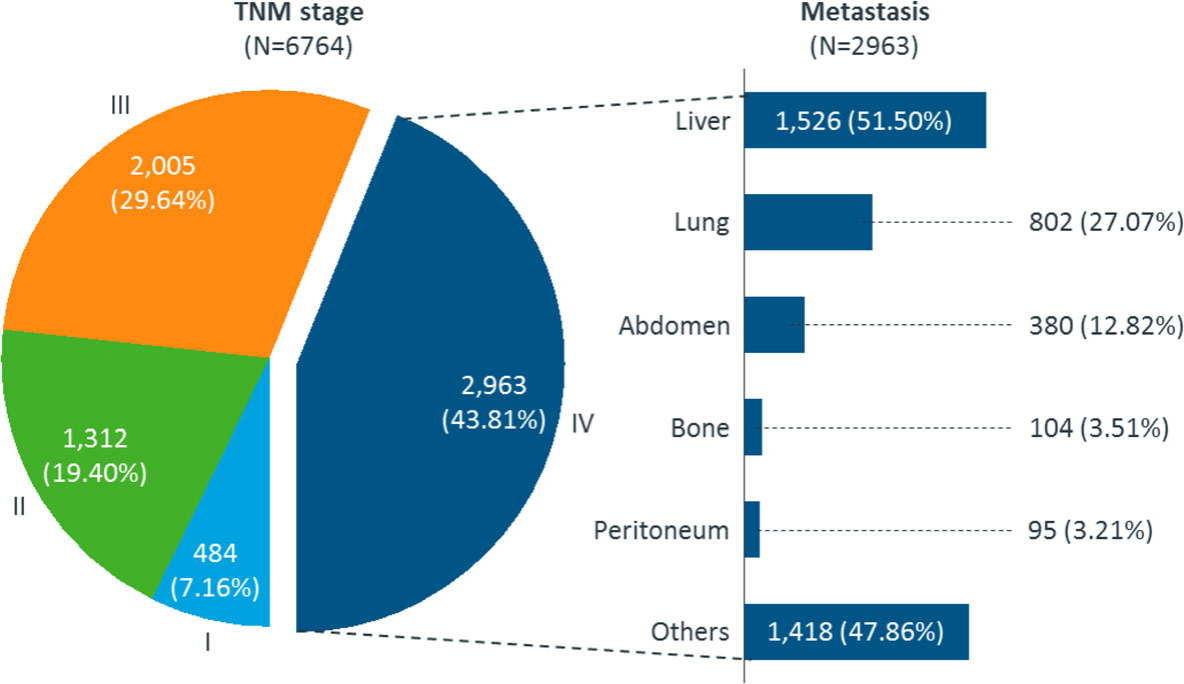
TNM stage and metastatic status at diagnosis

The mean age of CRC patients was 59 (SD 13) years old and 60% of them were males. Most of the patients (87%) presented in internal medicine departments. The size of primary tumor was only available among 36% (2926/8136) patients with a mean of 4.48 cm (SD 2.05). The KRAS mutation status testing rate was only 25%. The majority of CRC patients had left-sided primary tumor sites (71%) and were physically well (98%) at baseline with an Eastern Cooperative Oncology Group Performance Status (ECOG PS) scoring of 0 or 1 (Table 1). Of the 8136 CRC patients, 6764 (83%) had TNM classification records at primary diagnosis. Among them, 7.2, 19, 30 and 44% were at stages I, II, III and IV, respectively. Liver was the most common metastatic site (52%), followed by lung (27%) (Fig. 2).

**Table 1:** Baseline characteristics of CRC and mCRC patients

Characteristics	CRC patients	mCRC patients
	(*N* = 8136)	(*N* = 3878)
Age at diagnosis, year		
Mean (SD)	59 (13)	57 (12)
Median (IQR)	60 (50–68)	58 (49–66)
Min - max	18–96	18–96
Age group, n (%)		
18–49	1883 (23%)	1075 (28%)
50–59	2035 (25%)	1067 (28%)
60+	4218 (52%)	1736 (45%)
Gender, n (%)		
Female	3233 (40%)	1472 (38%)
Male	4903 (60%)	2406 (62%)
Department of practice, *n* (%)		
Internal medicine	7074 (87%)	3584 (92%)
Surgery	1062 (13%)	294 (7.6%)
Hospital geographic region, *n* (%)		
North	489 (6%)	416 (11%)
South	4972 (61%)	2258 (58%)
East	533 (6.6%)	463 (12%)
Midwest	2142 (26%)	741 (19%)
Primary tumor size at diagnosis, centimeters		
Missing, *n* (%)	2926 (36%)	2116 (55%)
Mean (SD)	4.48 (2.05)	4.52 (2.09)
Median (IQR)	4 (3–5.5)	4 (3–5.5)
Mutation status, n (%)		
KRAS MUT	794 (10%)	525 (14%)
KRAS WT	1246 (15%)	805 (21%)
Unknown	6096 (75%)	2548 (66%)
Primary tumor site at diagnosis, *n* (%)		
Left-sided	5751 (71%)	2659 (69%)
Right-sided	1767 (22%)	879 (23%)
Colorectal NOS	618 (7.6%)	340 (8.8%)
ECOG PS at diagnosis, *n* (%)		
Missing, n	2386	1470
0	724 (12%)	429 (18%)
1	4912 (86%)	1919 (80%)
2	65 (1.1%)	51 (2.1%)
3	11 (0.19%)	7 (0.29%)
4	4 (0.07%)	2 (0.08%)

Note: *CRC* colorectal cancer, *mCRC* metastatic colorectal cancer, *SD* standard deviation, *IQR*: interquartile range, *min* minimum, *max* maximum, *MUT* mutation, *WT* wild-type, *NOS* not otherwise specified, *ECOG PS* Eastern Cooperative Oncology Group Performance Status

### Disease recurrent risks for CRC patients with radical surgeries

**Fig. 3:**
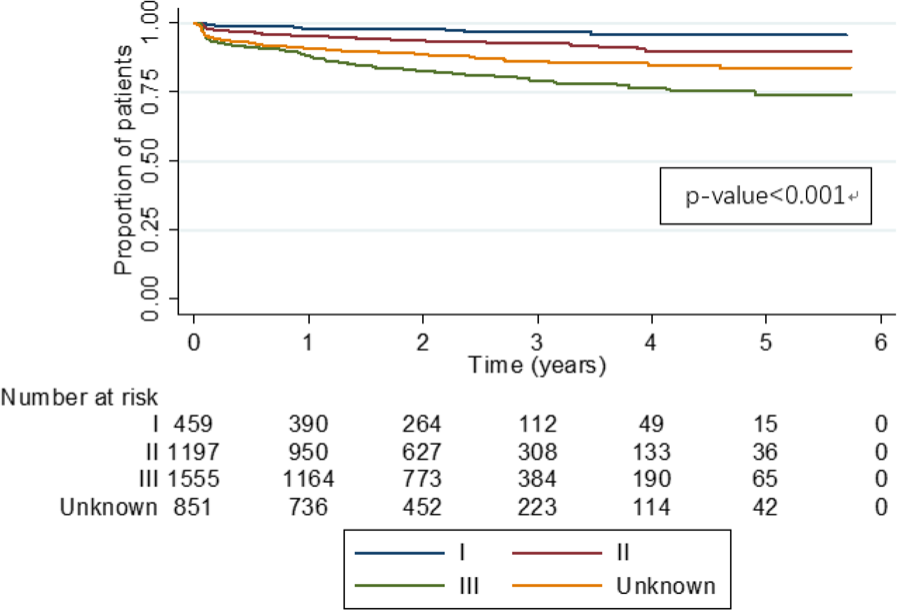
Disease-free survival curves from radical surgeries by TNM stage at diagnosis

For patients who underwent radical surgeries, the cumulative recurrence rate at year 1, 2, and 3 was 8.9, 16 and 30%, respectively. By different baseline TNM stage, an upward trend could be seen from baseline stage I to stage III for all 1-, 2- and 3-year recurrence rates (*p* < 0.001, Additional file 1: Table S1). Recurrence rates did not differ significantly between left- and right-sided primary tumor sites (Additional file 1: Table S2). The log-rank test showed that differences across TNM stage-specific, disease-free survival curves were of statistical significance (*p* < 0.001, Fig. 3).

### Baseline characteristics for mCRC patients

The mean age of mCRC patients at the primary diagnosis date were 57 years old (SD 12). There were more males in mCRC patients (62%) and most (92%) presented in internal medicine departments. The mean size of the primary tumor was 4.52 cm (SD 2.09). The KRAS mutation status testing rate was 35%. The majority of CRC patients had left-sided primary tumor sites (69%) and were physically well (98%) at baseline (Table 1).

### Treatment patterns for mCRC patients with palliative chemotherapy

**Fig. 4:**
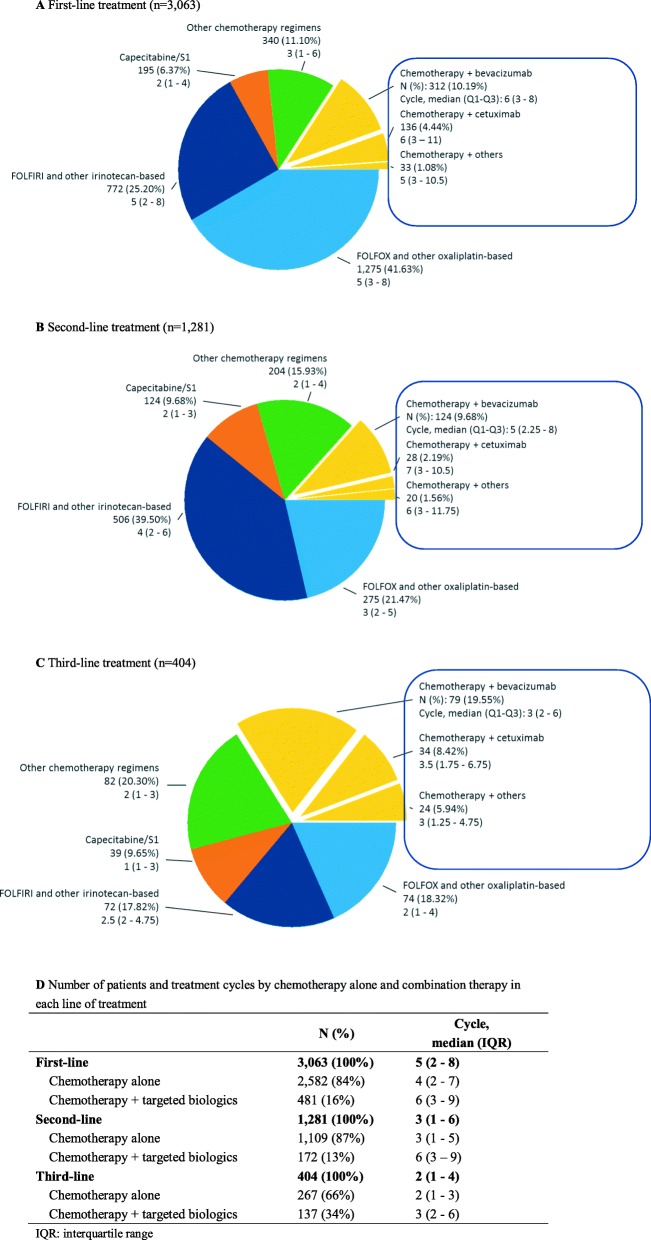
Treatment patterns of palliative chemotherapy for mCRC patients

Among the 3878 mCRC patients, 79% (3063) had records on first-line treatment of palliative chemotherapy, 1281 had records on second-line treatment, and 404 had records on third-line treatment. Fluorouracil, leucovorin, and oxaliplatin (FOLFOX) and other oxaliplatin-based regimens were the most frequently administered (1275/3063, 42%) in first-line, followed by fluorouracil, leucovorin, and irinotecan (FOLFIRI) and other irinotecan-based regimens (25%) (Fig. 4a). Usage of FOLFIRI and other irinotecan-based regimens increased remarkably to 40% in second-line, which dominated treatment of this line (Fig. 4b). Correspondingly, FOLFOX and other oxaliplatin-based regimens decreased to 21% in second-line. Less than one-sixth of patients received targeted biologics in combination with chemotherapy (16 and 13% in first- and second-line, respectively). Among these patients, the majority received bevacizumab (312/418, 75% and 124/162, 77% in first- and second-line, respectively). The proportion of patients receiving combination therapy with targeted biologics increased dramatically to 34% in third-line treatment (137/404), and bevacizumab was still the dominant choice (79/137, 58%) (Fig. 4c).

Treatment duration decreased in later lines of treatment. Median cycles were 5 in first-line, 3 in second-line and reduced to 2 in third-line treatment. In addition, patients receiving combination therapy with targeted biologics had longer median cycles than chemotherapy alone in all three lines of treatment (6 vs. 4, 6 vs. 3 and 3 vs. 2 in the first-, second- and third-line, respectively) (Fig. 4d).

### Treatment sequences for mCRC patients with palliative chemotherapy

In total, 1200 patients had records on both first- and second-line of treatments, of whom 404 patients had records on third-line of treatments.

Four hundred and ninety-five patients who received FOLFOX and other oxaliplatin-based regimens in first-line treatment changed to second-line, accounting for 39% (495/1275) of all patients starting treatment with this type of regimen. Among them, the majority (286/495, 58%) changed to FOLFIRI and other irinotecan-based regimens, which was also the most common sequence between first- and second-line treatments (286/1200, 24%). FOLFOX and other oxaliplatin-based regimens were somehow re-introduced to a small proportion of these patients (65/495, 13%) in second-line treatment.

The second most common sequence between first- and second-line treatments was moving from FOLFIRI and other irinotecan-based regimens to FOLFOX and other oxaliplatin-based regimens (128 out of 1200, 11%).

A total of 994 patients receiving chemotherapy alone in first-line treatment proceeded to second-line, accounting for 39% of all those received chemotherapy alone. One in ten of them (101/994, 10%) received targeted biologics in combination with chemotherapy as their second-line treatment.

**Fig. 5:**
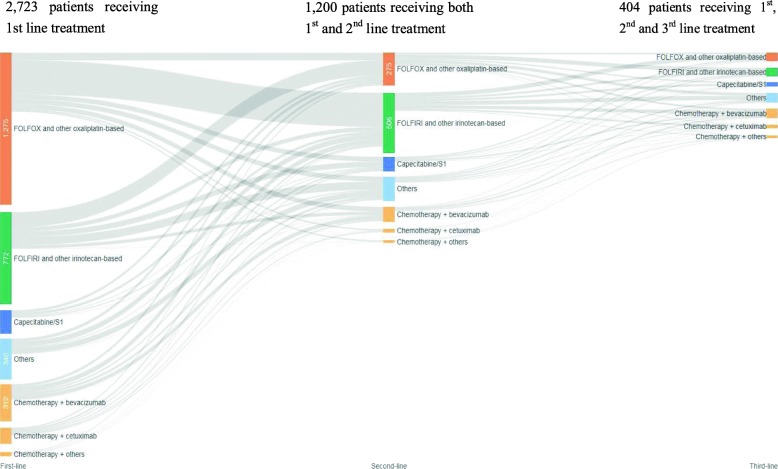
Treatment sequences of palliative chemotherapy for mCRC patients

Among the 275 patients receiving the dominant treatment choice in second-line, i.e., FOLFIRI and other irinotecan-based regimens, 151 (55%) proceeded to third-line. The most common sequence from the second-line was to FOLFOX and other oxaliplatin-based regimens (36/151, 24%), followed by to other chemotherapy alone (34/151, 23%). A total of 354 patients receiving chemotherapy alone in second-line proceeded to third-line, accounting for 32% of all those received chemotherapy alone. Among these 354 patients, over a quarter (105/354, 30%) added targeted biologics on top of chemotherapy, which also constituted a large proportion of total patients receiving combination therapy in this line of treatment (105/137, 77%). Figure 5 demonstrates treatment sequences of palliative chemotherapy from first- to third-line treatments.

## Discussion

To our knowledge, this is the first nationwide large-scale study on CRC and mCRC patient characteristics and treatment sequences in China. We described patient demographics and clinical characteristics, which provided a comprehensive and updated picture of Chinese CRC and mCRC patients in China. Analysis results on prognosis after radical surgeries and clinical practice in palliative chemotherapy can be used as real-world evidence to inform the management of CRC and mCRC. Importantly, our results demonstrated no dominant choice in the third-line therapy. This reflects a lack of consensus on the choice of third-line therapy in China and an urgent need to develop national guidelines on clinical practice.

In this study, the mean age of CRC and mCRC patients diagnosed during 2011 to 2016 was 59 and 57 years old, respectively. For both CRC and mCRC patients, there were more male CRC patients than females. These findings are not surprising compared to previous published reviews and regional studies in China [7–9]. It has been reported that estrogen could prevent CRC [23], which may explain the relatively small proportion of female patients.

In this study, approximately 30% of patients presented with TNM stage III and about 40% presented with TNM stage IV at diagnosis. The percentage of patients diagnosed with TNM stage III is generally in line with previous findings which reported a range of 30–40%; while, the percentage of patients identified with stage IV is larger than that reported in previous studies which is ranged 20–33% [24, 25]. The inconsistence on the percentage of patients diagnosed with stage IV across studies may be due to the differences in study samples, e.g. the current study included higher proportion of patients aged 60 years and over than other studies. It is also possible that the early detection of colorectal cancer in China is not as prevalent as in other countries. A decrease in the proportion of patients with advanced stage CRC may be expected if an early detection program can be implemented [24].

Most patients in this study did not have their KRAS mutation status tested. Although KRAS mutation analysis may provide additional useful information on risk stratification in colorectal cancer, the predictive value of KRAS mutation for non-response to chemotherapy is still questioning [23]. Previous studies found that the expression of KRAS is associated with recurrence, survival and benefit of adjuvant chemotherapy [26]. Future studies on the predictive value of KRAs in Chinese CRC patients are required.

As expected, the proportion of right-sided CRC (22%) found in this study was higher than that reported in the 1990s (15%) and 1980s (11%) [11]. A similar rightward shift in the primary tumor site of CRC has also been reported in North America, the United Kingdom, Japan and Northern Ireland [27–31], and it is associated with aging [32]. Our study has also found that among patients after radical surgeries, the primary tumor site was not associated with disease-free survival. This is supported by the finding from a most recent study which included 4426 Chinese patients with stage I, II and III CRC [33]. Patient at earlier stages had a significant improvement in survival. This finding underlines the importance of early diagnosis and increased awareness of CRC in China [34, 35].

Our study found that FOLFOX and other oxaliplatin-based regimens dominated first-line while FOLFIRI and other irinotecan-based regimens dominated second-line. This is consistent with findings from other studies. For example, a study with 1655 adult mCRC patients in the US reported that about 40% of patients received FOLFOX in first-line therapy, and about 26% of patients received FOLFIRI in second-line therapy [15]. Another US based study reported similar findings [36]. As to third-line therapy, there was no dominated treatments were identified in China. Importantly, targeted biologics were not frequently used in China until third-line treatment, treatment cycles in third-line was short and some patients moved back to their previously used therapy. In contrast, in the US, the most common third line treatment regimens are EGFR-containing therapies, such as combination of cetuximab and irinotecan, panitumumab or cetuximab monotherapy [15]. In other countries, such as Canada, although oxaliplatin and irinotecan were also the most common chemotherapy backbones for first- and second-line, chemotherapy was usually not used alone [17, 18]. This may imply poor treatment outcomes of targeted therapy in late lines, and a lack of consensus on both the timing and options for third-line treatment during the study period (2011–2016). Moreover, the current study found that a small proportion of patients treated with FOLFOX in the first-line setting either shifted to capecitabine/S1 or continued to use FOLFOX in the second-line setting, which was controversial to the guidelines for standard of care [37]. This gap between clinical practice and guidelines may be due to patients’ preference, economic status, physicians’ decisions [37–39]. Implementing education program and providing training courses to physicians as well as raising the public awareness of CRC might help with the treatment decision-making.

There have been some major changes in mCRC treatment, especially in the third line since 2017. For example, Bevacizumab (Avastin®) has been included into the national reimbursement list and Regorafenib (Stivarga®) has been approved by CFDA. On September 5th, 2018, Fruquintinib (Elunate®) was approved in China’s market. These new treatment options on market have increased regimen choice and likely expenditures. Despite of that, the optimal use of these agents along with chemotherapy needs further investigation as a phase II multicenter trial reported little objective responses to the combination of bevacizumab and chemotherapies among advanced CRC patients [40]. Further studies on the cost and effectiveness of treatment sequences using real-world data are warranted to provide important and updated evidence for improving patients’ survival.

This study had several limitations. Although we had a wide geographic coverage, all selected hospitals were tertiary hospitals located in large cities. In the three-tier healthcare system of China, patients with severe diseases, such as cancer, would mostly be referred to tertiary hospitals in large cities, but nevertheless those who cannot afford such treatments may present elsewhere. Therefore, sampling bias may still present a barrier to understanding the complete picture of CRC and mCRC patients in China. In addition, if patients visited hospitals outside the selected ones during the observation period, those records would not have been captured and therefore could not be analyzed in this study. Moreover, we utilized a multi-center database specialized in oncology, however the study population was a sample of the whole CRC patient population seen for care in the 12 hospitals. There is no evidence that the sampling process and data collection may introduce significant selection bias. Some key time points, such as the appearance of metastasis, were not recorded in EMRs and were estimated using best proxies. Finally, some key information might not be available or well documented in EMR databases in China. For examples, death information was poorly documented and disease progression information was not directly captured. It is not possible to link an EMR database to the National Death Registry to obtain Deaths. Information on confounders, for examples, diet, smoking, alcohol, was not available in the database. Thus, in the current study, we were not able to provide a more comprehensive view of effectiveness of regimens on survival. It is evident that further real-world evidence, especially from Registries or prospective studies, is required.

## Conclusions

In this multi-center, retrospective study of patients with CRC and mCRC, the current status of CRC and mCRC clinical management were investigated. Among mCRC patients, FOLFOX and FOLFIRI were the dominated first-line and second-line therapies, respectively. There was an increasing trend of using targeted biologics in third-line therapy. With new medications approved or included in national reimbursement scheme in China in recent years, the current findings will be useful in exploration of changing trends of therapies for CRC patients in China.

## Supplementary information

**Additional file 1: Table S1.** Recurrent rates from radical surgeries by TNM stage at diagnosis. **Table S2.** Recurrent rates from radical surgeries by primary tumor site

## Data Availability

The data underlying the study is from EMR databases in multiple tertiary hospitals in China. It is not publicly available, and restrictions apply to the availability of the data.

## Abbreviations

ASIRC: Age-standardized incidence rate by Chinese standard population
CRC: Colorectal cancer
ECOG PS: Eastern Cooperative Oncology Group Performance Status
EMRs: Electronic medical records
FOLFIRI: Fluorouracil, leucovorin and irinotecan
FOLFOX: Fluorouracil, leucovorin and oxaliplatin
IQR: Interquartile range
Max: Maximum
mCRC: Metastatic colorectal cancer
Min: Minimum
MUT: Mutation
NCCR: National central cancer registry
NOS: Not otherwise specified
SD: Standard deviation
TNM: Tumor, models and metastases
WT: Wild-type
