# Supplementary material for: Disease characteristics and treatment patterns of Chinese patients with metastatic colorectal cancer: a retrospective study using medical records from China
Source: BMC Cancer. 2020 Feb 18;20:131. doi: 10.1186/s12885-020-6557-5 (PMC7029588; doi:10.1186/s12885-020-6557-5)
Supplement: Supplementary file 1 — Additional file 1: Table S1. Recurrent rates from radical surgeries by TNM stage at diagnosis. Table S2. Recurrent rates from radical surgeries by primary tumor site [file 12885_2020_6557_MOESM1_ESM.docx]

**Table S1** Recurrent rates from radical surgeries by TNM stage at diagnosis

| **1-year recurrence rate (N=3,556)** | | | | | | |
| --- | --- | --- | --- | --- | --- | --- |
| **Recurrence, n (%)** | **Stage I** | **Stage II** | **Stage III** | **Stage unknown** | **p-value** | **Total** |
|  | **(n=398)** | **(n=1,003)** | **(n=1,342)** | **(n=813)** |  | **(n=3,556)** |
| Yes | 8 (2.0%) | 53 (5.3%) | 179 (13%) | 78 (9.6%) | <0.001 | 318 (8.9%) |
| No | 390 (98%) | 950 (95%) | 1,163 (87%) | 735 (90%) |  | 3,238 (91%) |
| **2-year recurrence rate (N=2,525)** | | | | | | |
| **Recurrence, n (%)** | **Stage I** | **Stage II** | **Stage III** | **Stage unknown** | **p-value** | **Total** |
|  | **(n=273)** | **(n=695)** | **(n=1,014)** | **(n=543)** |  | **(n=2,525)** |
| Yes | 9 (3.3%) | 68 (9.8%) | 241 (24%) | 91 (17%) | <0.001 | 409 (16%) |
| No | 264 (97%) | 627 (90%) | 773 (76%) | 452 (83%) |  | 2,116 (83%) |
| **3-year recurrence rate (N=1,476)** | | | | | | |
| **Recurrence, n (%)** | **Stage I** | **Stage II** | **Stage III** | **Stage unknown** | **p-value** | **Total** |
|  | **(n=123)** | **(n=380)** | **(n=649)** | **(n=324)** |  | **(n=1,476)** |
| Yes | 11 (8.9%) | 73 (19%) | 265 (41%) | 101 (31%) | <0.001 | 450 (30%) |
| No | 112 (91%) | 307 (81%) | 384 (59%) | 223 (69%) |  | 1,026 (70%) |

P-values were calculated from Fisher's exact tests.

**Table S2** Recurrent rates from radical surgeries by primary tumor site

| **1-year recurrence rate (N=3,556)** | | | | | |
| --- | --- | --- | --- | --- | --- |
| **Recurrence, n (%)** | **Left-sided** | **Right-sided** | **Colorectal NOS** | **p-value** | **Total** |
|  | **(n=2,609)** | **(n=714)** | **(n=233)** |  | **(n=3,556)** |
| Yes | 225 (8.6%) | 67 (9.4%) | 26 (11%) | 0.363 | 318 (8.9%) |
| No | 2,384 (91%) | 647 (91%) | 207 (89%) |  | 3,238 (91%) |
| **2-year recurrence rate (N=2,525)** | | | | | |
| **Recurrence, n (%)** | **Left-sided** | **Right-sided** | **Colorectal NOS** | **p-value** | **Total** |
|  | **(n=1,848)** | **(n=519)** | **(n=158)** |  | **(n=2,525)** |
| Yes | 296 (16%) | 81 (16%) | 32 (20%) | 0.349 | 409 (16%) |
| No | 1,552 (84%) | 438 (84%) | 126 (80%) |  | 2,116 (84%) |
| **3-year recurrence rate (N=1,476)** | | | | | |
| **Recurrence, n (%)** | **Left-sided** | **Right-sided** | **Colorectal NOS** | **p-value** | **Total** |
|  | **(n=1,052)** | **(n=311)** | **(n=113)** |  | **(n=1,476)** |
| Yes | 321 (31%) | 87 (28%) | 42 (37%) | 0.193 | 450 (30%) |
| No | 731 (69%) | 224 (72%) | 71 (62%) |  | 1,026 (70%) |
